# Hemodialysis‐associated radioactive waste management in [^131^I]I and [^177^Lu]Lu radionuclide therapy

**DOI:** 10.1002/acm2.70314

**Published:** 2025-10-24

**Authors:** Dennis Kupitz, Heiko Wissel, Martin Volk, Michael C. Kreissl, Oliver S. Grosser

**Affiliations:** ^1^ Department for Radiology and Nuclear Medicine University Hospital Magdeburg Magdeburg Germany; ^2^ Research Campus Stimulate Otto‐von‐Guericke University Magdeburg Germany

**Keywords:** hemodialysis, nuclear medicine, radiation protection, radionuclide therapy

## Abstract

**Background:**

Radionuclide therapy, such as with iodine‐131 or lutetium‐177–labeled substances, is used to treat thyroid cancer and neuroendocrine tumors. Due to the incorporated radioactivity, patients must remain hospitalized until radiation levels are deemed safe, according to national regulations. This is particularly problematic for patients who require hemodialysis (HD). While contamination of dialysis waste after radionuclide therapy is generally well recognized, a critical gap remains regarding its duration and extent. This poses risks for patient management and outpatient staff.

**Purpose:**

This study aims to demonstrate the problems with classifying residual waste and provides arguments for appropriate regulation. It evaluates if the radioactivity of the dialysate and residuals at discharge, based on whole‐body measurements, complies with relevant limits. If not, the waste is classified as radioactive and treated accordingly.

**Methods:**

We studied four patients treated by radionuclide therapy who required dialysis: three received [^131^I]I for thyroid disease and one [^177^Lu]Lu‐DOTATATE for neuroendocrine neoplasia. Dialysis was performed in the nuclear medicine ward, and patients were discharged once the local dose rate limits were met. Subsequent dialysis sessions were conducted in a radiation protection area until the activity concentration in the dialysate dropped below the specific limit.

**Results:**

Patient 1 ([^131^I]I, thyroid cancer) showed the highest extraction rate and was discharged after 9 days, requiring nine additional dialysis sessions over 21 days. Patients 2 and 3 ([^131^I]I, benign thyroid disease) were discharged after 8 and 4 days, with dialysis continued for 31 and 18 days under supervision, respectively. Patient 4 ([^177^Lu]Lu‐DOTATATE) lost 4.9% of the total body radioactivity per dialysis session and was discharged after 9 days; supervised dialysis continued for 17 days.

**Conclusions:**

This study highlights the need for refined monitoring and the limitations of standard discharge strategies for patients requiring dialysis during radionuclide therapy. Even if dose limits are met, dialysate and residuals may still require regulated handling and storage.

## 1 INTRODUCTION

Nuclear medicine procedures involve the use of radiopharmaceuticals for both diagnostic and therapeutic applications.

The most commonly administered therapeutic radionuclide is radioiodine ([^131^I]I).^1^ Radioiodine therapy is used to treat hyperthyroidism (such as toxic adenomas and Graves’ disease), as well as goiter.^2^, ^3^ Furthermore, radioiodine therapy is a standard post‐surgical treatment for many patients with differentiated thyroid cancer.^4^, ^5^

For other tumors, radionuclide therapy involves the administration of radiolabeled peptides or antibodies (such as radiolabeled somatostatin [^177^Lu]Lu‐DOTATATE)^6^ or ligands that bind to prostate‐specific membrane antigen ([^177^Lu]Lu‐PSMA‐617).^7^

According to radiation protection regulations in certain countries, patients receiving nuclear medicine therapy must remain hospitalized for a specified duration. Discharge is only permitted if the dose rate falls below a predefined limit. This limit is based on the requirement that people in the general population should not be exposed to more than a certain effective dose per year. The ICRP proposes a public effective dose limit of 1 mSv per year,^8^ which has been adopted by the European directive, for example.^9^ Other countries permit higher values; for example, 5 mSv in the USA. However, patient‐specific behavioral instructions must be given if the dose will exceed 1 mSv.^10^

Several factors must be considered when managing patients during and after radionuclide therapy. These include evaluating various exposure scenarios to establish radiation exposure limits for staff, the general public, and the environment (including direct irradiation from treatment activity absorbed by the patient or from radioactive excretions). Depending on national regulations, radioactive waste and excretions must be collected and stored for decay during hospitalization (e.g., via a dedicated wastewater management system).

For patients with normal renal function, a substantial proportion of therapeutic activity is excreted in the urine and feces during hospitalization. In patients with thyroid disease, >75% of the administered [^131^I]I activity is excreted by the kidneys within 24–36 h, depending on tumor burden.^11^ For patients treated with [^177^Lu]Lu, up to 50% of the administered activity is excreted within 6 h, assuming normal renal function.^12^, ^13^

For patients with impaired renal function requiring hemodialysis (HD), treatment protocols must be adjusted to mitigate radiation exposure from the dialysate.^14^, ^15^, ^16^

Although the extraction of radioactive (labeled) substances by HD was recognized in principle, there is a lack of systematic understanding or guidance regarding the handling of activity extracted by HD.^17^, ^18^, ^19^ For example, there is no guidance on waste management following HD in radionuclide therapy.

This study analyzed patients who required HD after radionuclide therapy. Standard whole‐body dose rate (WBDR) measurements were performed to evaluate public exposure. Additionally, the extracted activity and activity concentration of the dialysate were examined until the national limit for unrestricted handling was reached. Accordingly, case‐based proposals for managing radiation protection tasks were provided according to national regulations, while the findings also offer insights applicable to staff safety, contamination control, and radioactive waste management in other institutional and regulatory contexts.

## 2 METHODS

For this study, the data of all patients (*n* = 4; Table 1) with impaired renal function on HD treated between June 2018, and March 2024, in our department for radionuclide therapy were examined. All patients provided written informed consent for the evaluation of their data, and approval was obtained from the Institutional Ethics Committee (registration number: 36/25). The first patient (Patient 1) was diagnosed with papillary thyroid carcinoma and received adjuvant treatment with 3.996 GBq [^131^I]I after total thyroidectomy. During follow‐up, two iodine whole‐body scintigraphy scans were performed using diagnostic activities of 199 MBq and 187 MBq [^131^I]I (Table 1; Patient 1a and b). Patient 2 was treated for nodular goiter with 519 MBq [^131^I]I, and Patient 3 was treated for a toxic nodule with 416 MBq [^131^I]I. Patient 4 began [^177^Lu]Lu‐DOTATATE therapy for the treatment of neuroendocrine neoplasia of the terminal ileum with a reduced dose of 3.71 GBq [^177^Lu]Lu.

**TABLE 1:** Patient and therapy parameters/characteristics.

Patient no.	Indication	Radio‐ nuclide	Injected activity [MBq]	First dialysis [h]	Ward stay [days]	WBDR for discharge from ward (in 2 m) [µSv/h]	Super‐vision after release from ward [days]	Dialysate activity^a^ [MBq]	Dialysate activity concentration^a^ [kBq/g]	#HD^b^	HD container storage time [days]
1	Papillary thyroid carcinoma (therapy)	[^131^I]I	3996	72	9.1	1.2	12	130	5.1	9(5)	69
1a	1. Whole‐body scintigraphy	[^131^I]I	199	24	2.1	1.0	11	–	–	6(5)	69
1b	2. Whole‐body scintigraphy	[^131^I]I	187	48	2.1	1.8	14	–	–	7(6)	65
2	Struma multinodosa [150 Gy]	[^131^I]I	519	24	8.0	3.5	23	4	0.2	13(10)	53
3	Thyroid autonomy [150 Gy]	[^131^I]I	416	24	4.1	3.2	14	8	0.4	8(6)	41
4	NEN Ileum	[^177^Lu]Lu	3707	24	9.0	1.4	11	127	6.9	9(5)	45

Abbreviation: WBDR, whole‐body dose rate.

^a^ At the time when discharge would generate 1 mSv/year in the population because of the WBDR.

^b^ Total number of hemodialysis (HD) cases analyzed (in parentheses, the number of HD sessions after patient discharge from the specialized ward until reaching the limit for unrestricted handling).

After administration, the first dialysis was performed in the nuclear medicine therapy ward. Once the respective local WBDR thresholds were met to ensure compliance with the 1 mSv/year national limit for the general population, the patients were discharged home. For this purpose, all WBDR measurements were performed using a calibrated measuring device (model FH 40 GL‐10, ambient dose equivalent rate meter, Thermo Fisher Scientific Messtechnik GmbH, Germany), which measures gamma and X‐rays in the range of 0.5 µSv/h to 100 mSv/h. For patients treated with [^131^I]I, discharge is permitted only after the WBDR fell below 3.5 µSv/h at a distance of 2 m.^20^ This corresponds to a incorporated whole‐body activity of 250 MBq.^20^ For patients treated with [^177^Lu]Lu‐PSMA in Germany, the recommended WBDR depends on the number of treatment cycles per calendar year. It is 4.3 µSv/h at a distance of 2 m for one treatment cycle per year (equivalent to 2300 MBq whole‐body activity), or 1.1 µSv/h for four cycles per year.^21^

Further dialysis was performed under the supervision of the Nuclear Medicine Department. The dialysis schedule was not synchronized with the day of radiopharmaceutical administration; thus, patients received their first HD at different time points after treatment (Table 1). HD was performed using a 4 mmol/L potassium solution for hemofiltration (multiBic, Fresenius Medical Care AG, Bad Homburg, Germany) and capillary hemofilters for continuous renal replacement therapy (model AV 600S, Fresenius Medical Care AG, Bad Homburg, Germany). These devices and configurations are standard equipment and configurations used for mobile dialysis, regardless of whether radiopharmaceuticals have been administered previously.

The total activity and activity concentration extracted during HD were analyzed for each session. HD was performed under the supervision of the Nuclear Medicine Department and evaluated for the study until the level for unrestricted disposal of radioactive waste, as defined by national radiation protection regulations, was reached (defined by 1 MBq and 10 Bq/g for [^131^I]I, and 10 MBq and 100 Bq/g for [^177^Lu]Lu).^22^ The filtration rate varied between 2500 and 4500 mL/h, with ultrafiltration between –1200 and –400 mL. During each HD, the dialysate was collected in two 10 L bags. For each bag, activity concentration (one sample per bag) and dialysate volume were measured. The two dialysate bags and other residual materials from dialysis (such as tubes and gloves) were collected in standard 50 L waste containers (one residual container per HD) and stored until release.

Dialysate samples (3 mL each) were measured using a commercial well counter equipped with an NaI scintillation detector (model ISOMED 2100, multichannel analyzer, Nuvia Instruments GmbH, Germany). The energy window for the [^131^I]I samples was set to 370 ± 55 keV, and for the [^177^Lu]Lu samples, it was 208 ± 30 keV. Each measurement was performed with an integration time of 60 s and the background counts were determined for each measurement. The measured counts were converted into activity values using previously determined calibration factors. The system undergoes regular quality control in accordance with International Electrotechnical Commission recommendations for gamma‐counting systems.^23^

## 3 RESULTS

**FIGURE 1:**
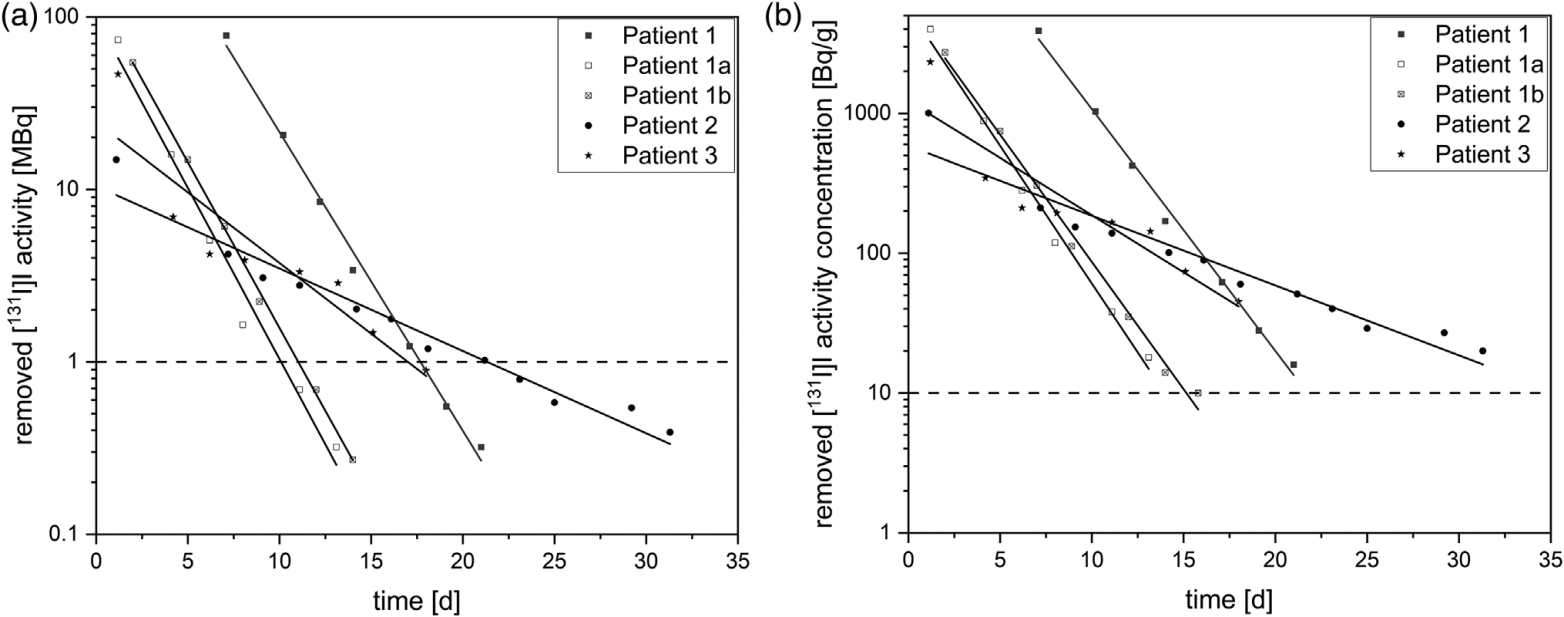
(a) Activity and (b) activity concentration extracted by hemodialysis (HD) for Patients 1–3 treated with [^131^I]I. The dashed horizontal line corresponds to the German national limit for unrestricted release of residual activity (A: 1 MBq and B: 10 Bq/g). Data from the first two HD sessions in Patient 1 were not available due to a technical issue.

The extracted [^131^I]I activity and corresponding activity concentrations from dialysis for Patients 1–3 are shown in Figure 1a and b.

Across all dialysis sessions, the extraction rate for Patient 1 was –7.21 kBq/h for therapeutic administration and –8.25 kBq/h and –8.73 kBq/h (all R^2^ ≥ 0.98, R^2^—coefficient of determination for regression analysis) for the two diagnostic administrations used in the whole‐body imaging procedure. The activity concentration decreased at rates of –0.007 Bq/g/h, –0.008 Bq/g/h, and –0.008 Bq/g/h, (all R^2^ = 0.99). For Patient 2, the extraction rate was –1.99 kBq/h (R^2^ = 0.96), and the activity concentration decreased at a rate of –0.002 Bq/g/h (R^2^ = 0.93). For Patient 3, the activity decreased by –3.41 kBq/h (R^2^ = 0.81) with a corresponding decrease in activity concentration of –0.003 Bq/g/h (R^2^ = 0.81). In all cases, the total extracted activity exceeded the corresponding national activity concentration limit.

Patient 1 could have been discharged from the ward after 6.5 days based on the WBDR. However, the patient remained in the ward until the ninth day due to medical reasons. In the follow‐up examinations (Patient 1a and Patient 1b; Table 1), the patient was discharged after only 2 days, with the WBDR already below 3.5 µSv/h at that time. However, the patient continued to undergo dialysis under the supervision of the nuclear medicine department until the activity values of the dialysate filtrate fell below the limit for exemption from regulatory control

**FIGURE 2:**
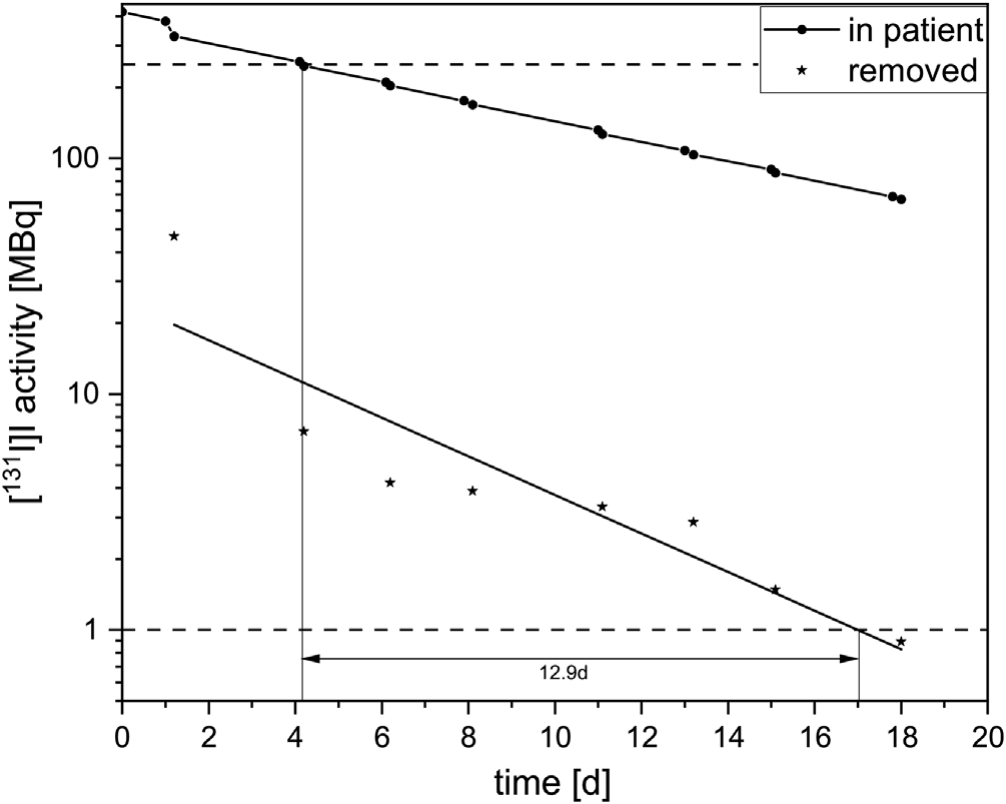
Comparison of residual [^131^I]I activity in the body and [^131^I]I activity removed by dialysis for Patient 3. The dashed horizontal lines correspond to the German national limits (250 MBq residual activity in the body and 1 MBq for unrestricted handling of [^131^I]I activity). The biological half‐life was calculated as T_1/2_ = 190.9 ± 15.4 h.

Figure 2 shows a comparison of the residual activity in the body and activity removed by dialysis in Patient 3. On the fourth day of hospitalization, the WBDR fell below the discharge limit. Simultaneously, 10 MBq [^131^I]I was removed via dialysis. Fourteen days after discharge and 18 days after treatment, the activity of the [^131^I]I removed by dialysis fell below the local exemption limit.

**FIGURE 3:**
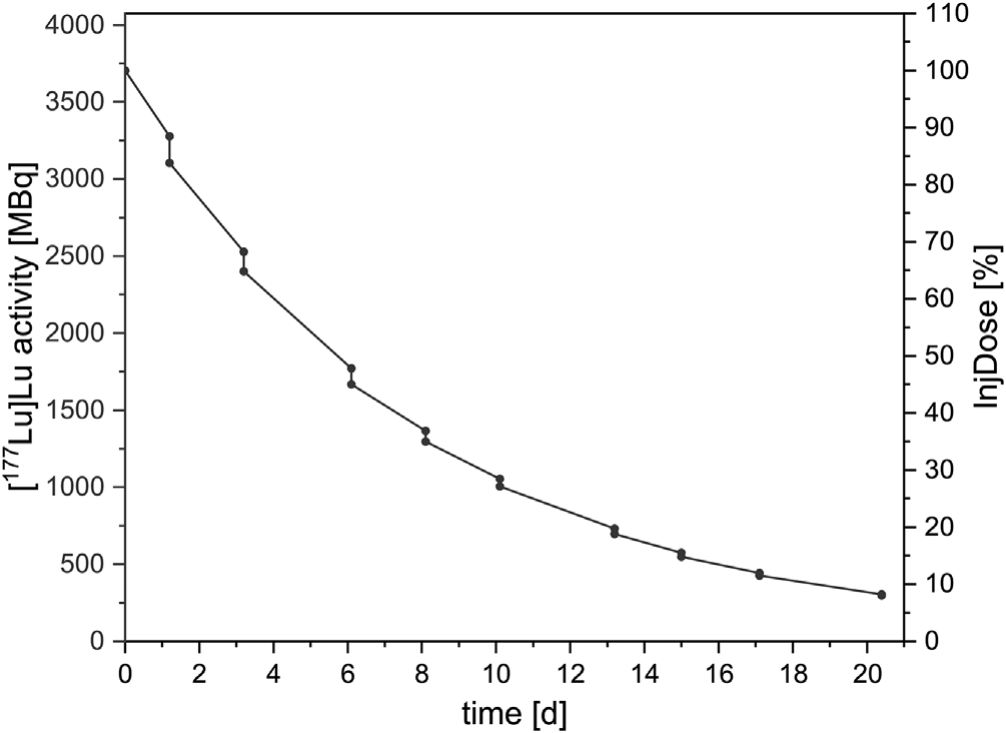
Activity course or % injected dose (InjDose) of Patient 4 after [^177^Lu]Lu application over a period of 20 days, during which a total of nine dialysis sessions were performed.

Figure 3 shows an example of the [^177^Lu]Lu excretion curve for Patient 4 until it fell below the national limit. Because of the lack of biological excretion, radioactivity in these patients decreases exclusively through physical decay. During dialysis, a portion of the activity is extracted, causing an abrupt reduction in total activity. For each dialysis session, an average of 4.9% of the injected dose (InjDose) was removed. After the ninth dialysis session, the extracted activity fell below the local exemption limit.

**FIGURE 4:**
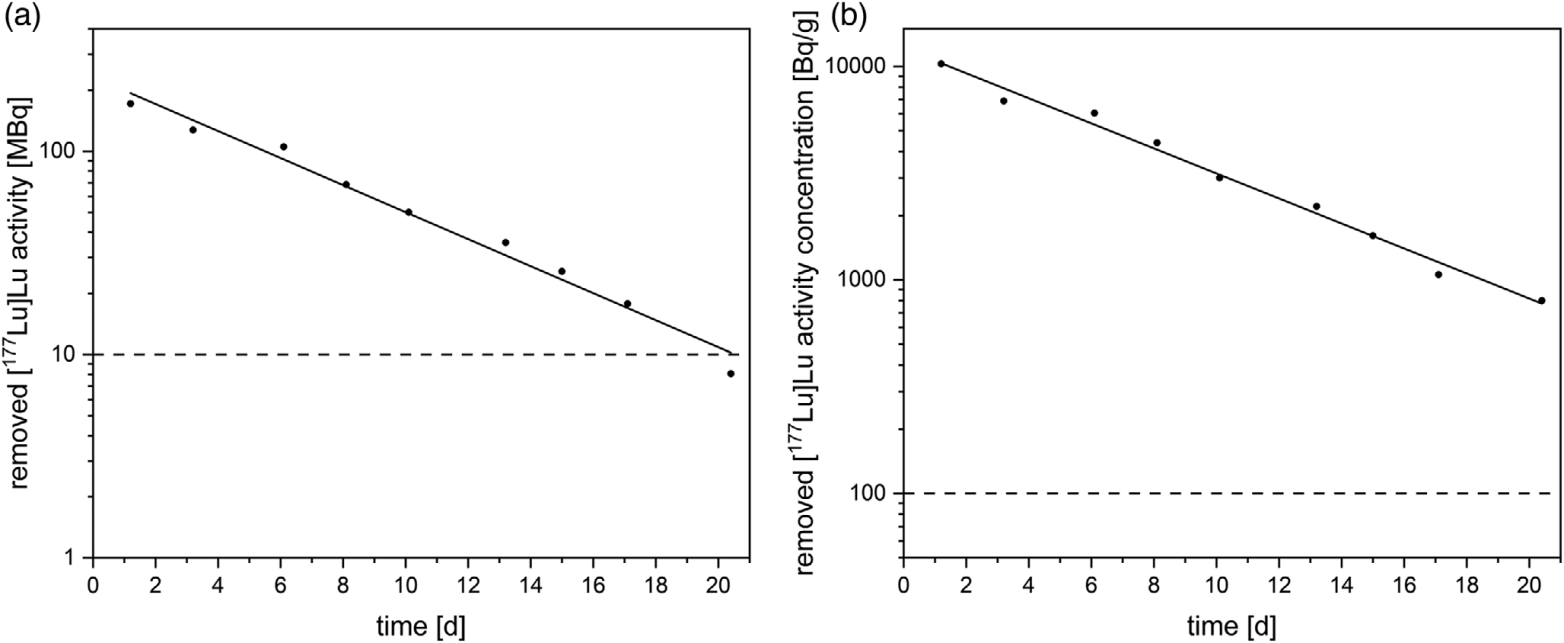
(a) Removed activity and (b) activity concentration of the fourth patient treated with [^177^Lu]Lu. The dashed horizontal line corresponds to the German national limit (A: 10 MBq and B: 100 Bq/g).

The [^177^Lu]Lu activity removed by dialysis gradually declined, from 172 MBq (10.3 kBq/g) to 8 MBq (0.8 kBq/g) (Figure 4a and b). The extraction rates for Patient 4 were –2.77 kBq/h (R^2^ = 0.98) and –0.002 Bq/g/h (R^2^ = 0.99).

The determined extraction rates for [^131^I]I activities differ among the observed patients and ranging from –1.99 to –8.73 kBq/h. The extraction rate for [^177^Lu]Lu activity was within the same range as the [^131^I]I values.

**FIGURE 5:**
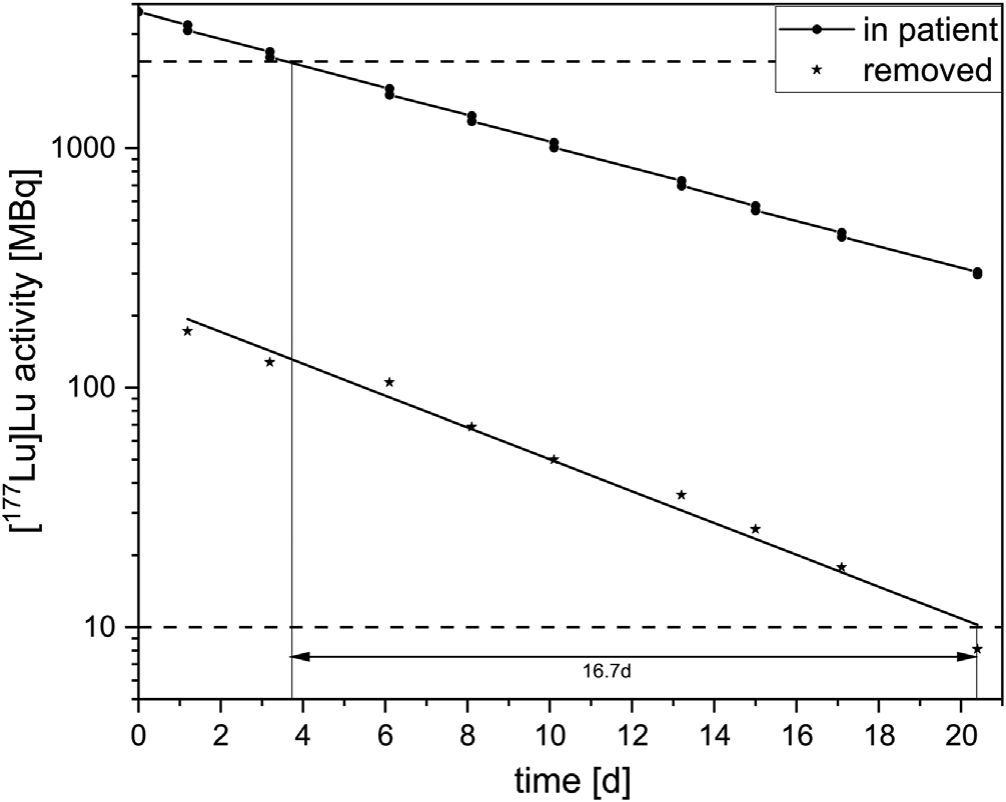
Comparison of residual [^177^Lu]Lu activity in the body and [^177^Lu]Lu activity removed by dialysis for Patient 4. The dashed horizontal lines correspond to the German national limits (2300 MBq residual activity in the body and 10 MBq for unrestricted handling of [^177^Lu]Lu activity).

Figure 5 shows a comparison of the residual activity in the body and activity removed by dialysis in Patient 4. On the third day of hospitalization, the patient's WBDR fell below the discharge threshold; whereas the extracted activity remained above the national limit for total activity by a factor of 12.7. However, for medical reasons, the patient remained hospitalized (Table 1).

## 4 DISCUSSION

In nuclear medicine, multiple aspects of radiation protection must be considered, including the monitoring and management of exposure for relatives, caregivers, the general population, and the environment. Key considerations include emissions from patients and radioactive excretions.

In countries where national regulations impose limits on radioactive excretions, waste is managed through a wastewater collection and storage system (e.g., during hospitalization). For patients with normal renal function, most of the activity is excreted during hospitalization in a specialized ward. After discharge, renal excretion typically occurs in the patient's private environment, which is generally considered safe from a radiation protection perspective. However, the management of excreted waste remains a critical aspect of environmental protection.

The presence of radioactively contaminated residues must not be overlooked. This includes dialysate. While the potential for radioactive contamination of dialysate is recognized in principle, there is a lack of systematic understanding or guidance regarding the persistence of such contamination over an extended period after radionuclide administration. Clinical practice often assumes that once the patient's WBDR falls below discharge thresholds, they can resume standard dialysis procedures in outpatient settings. However, in this study, we demonstrated that even when a patient meets the local WBDR threshold for discharge, residual dialysis materials may still exceed legal clearance values for disposal. Thus, the Nuclear Medicine Department remains responsible for handling this waste. Despite the limited number of cases, our cohort illustrates the clinical rarity and complexity of this scenario.

Our analysis is based on German radiation protection regulations, as our work was conducted within that national framework. However, we would like to emphasize that the underlying principles of radiation safety, such as minimizing exposure, controlling contamination, and managing radioactive waste—are universal and form the foundation of international radiological protection guidelines.

We demonstrated that the patients met the generally applied discharge criteria based on WBDR at a specific distance. However, their excretion may still contain radioactivity above permitted limits for activity or activity concentration. It can take several days or even weeks for the dialysate activity to fall below the corresponding limit after discharge. This creates a latent radiation risk for dialysis staff, suggesting the need for continued radiation protection planning even after hospitalization ends. This requires additional organizational efforts regarding space, personnel, and radiation protection and necessitates careful planning due to the complex management of both patients and radioactive waste. Furthermore, each dialysis process results in residual materials that must be stored until they can be properly disposed of after weeks or months of decay.

The excretion rate depends on the frequency and interval of dialysis, as well as the type of dialysis performed (e.g., HD or peritoneal dialysis). A small proportion of the activity can also be cleared via feces, sweat, and other bodily processes, so it varies greatly from person to person. However, in the case of terminal renal insufficiency, where there is no longer any significant natural urine excretion, there is an upper limit. In this case, the biological half‐life is equal to the physical half‐life. This is the case with Patient 3, for example. In this case, the half‐life is 190.9 h, which corresponds to the physical half‐life of iodine‐131 (see Figure 2). A shorter biological half‐life has been reported in case report following iodine‐131 treatment with peritoneal dialysis. In this case, the half‐life was 102 h.^24^ An individual comparison of the extraction rates in Patient 1 suggests that similar excretion can be expected for the second or third treatment, regardless of the applied activity. This may allow better planning for patients requiring multiple treatments.

Patients were permitted to leave the radiation protection area of the nuclear medicine ward once their WBDR fell below the threshold of 1 mSv/year for public exposure. For patients treated with [^131^I]I, discharge in Germany is only permitted after the WBDR has falls below 3.5 µSv/h at a distance of 2 m.^20^ This value may differ in other countries: the discharge limit for [^131^I]I is 20 µSv/h in France,^25^ 30 µSv/h in Italy,^26^ and 70 µSv/h in the USA,^11^ in each case measured at a distance of 1 m. Patients often fall below the local dose rate limit for WBDR after just a few days.

However, dialysis‐dependent patients receive further dialysis treatment in specialized facilities, rather than in private environments. Personnel working there, whether consciously or unconsciously, handle radioactive residues immediately after therapy without possessing the corresponding authorization to handle radioactive material depending on national regulations. The potential risk arises less from exposure to the residual activity present in the body than from the dialysate, which contains activities above the permitted limits and poses a contamination risk.

In German radiation protection law, there are threshold values for both activity (Bq) and activity concentration (Bq/g), with the limits for activity concentration being stricter than those for activity for the same radionuclide. Both limit values are valid, must be applied, and must be undercut for release. To decide if the patient's dialysis should continue to be monitored by personnel certified in radiation protection, we considered the dialysate as a single unit and used the activity limit (Bq) because it is stored in a bag and not discharged into the wastewater system. The stricter activity concentration limit (Bq/g) is used to determine the subsequent release of the 50 L containers that hold the dialysate bags and other residual materials, such as gloves, tubing, and drapes, from dialysis. Our data shows that, from a radiation protection perspective, the timeframe for managing dialysis‐related residues exceeds the hospitalization period by a significant amount. Therefore, a specific national implementation plan is required for managing patients and handling dialysate containing radioactive residues. Although additional efforts may be required in caring for dialysis‐dependent patients, this should not be interpreted as a disadvantage during treatment.

Dialysis machines have different techniques and requirements for patients, but most have core functions and can perform multiple tasks. Both stationary and mobile dialysis machines were used in nuclear medicine therapy. In stationary machines, the dialysate leaves the dialyzer and is collected in a closed circuit within the machine before being continuously discharged into the hospital wastewater system (or into a central storage tank) via a drain connection. This must be addressed under specific aspects for radioprotection purposes (e.g., the emission of radioactive isotopes). Mobile dialysis systems, by contrast, collect dialysate in bags rather than discharging it directly into the sewer system. These systems offer greater flexibility and allow dialysis to be performed in environments without direct access to a sewer system or where discharge is not desired. This approach enables controlled disposal and reduces the risk of contaminating wastewater and the environment. After dialysis, full dialysate bags are either disposed of by nursing staff or placed in an appropriate disposal system. Depending on national or local regulations, the dialysate is disposed of as hazardous medical waste or as part of the standard waste stream. Using bagged dialysate may result in additional costs and effort for bag replacement and disposal. However, no modifications were made to the dialysis system regarding radiation protection.

## 5 CONCLUSION

This work highlights the need for extended monitoring of dialysis patients undergoing radionuclide therapy. It demonstrates a discrepancy between WBDR based discharge workflows defined by national regulations and the practical requirements for managing radioactivity excreted during HD. Special attention must be given to the activity and activity concentration of the dialysate extracted by HD, as these values may still exceed the legal limit for unrestricted waste handling. When planning nuclear medicine procedures for these patients, it is important to consider both patient management, such as organizing dialysis within radiation protection area, and the handling and long‐term storage of radioactive residues.

## AUTHOR CONTRIBUTIONS

The conceptualization of this study was carried out by Dennis Kupitz and Oliver S. Grosser. The methodology was developed by Dennis Kupitz, Heiko Wissel, and Oliver S. Grosser, and the measurements were performed by Dennis Kupitz, Heiko Wissel, and Martin Volk. Data analysis, statistical evaluation, and visualization were conducted by Dennis Kupitz, Martin Volk, and Oliver S. Grosser. The original draft was written by Dennis Kupitz. The draft was reviewed and edited by all authors. Supervision was provided by Oliver S. Grosser and Michael C. Kreissl. Funding and resources were secured by Oliver S. Grosser and Michael C. Kreissl. All authors read and approved the final manuscript.

## CONFLICT OF INTEREST STATEMENT

The authors have no relevant conflicts of interest to disclose.

## Data Availability

The raw datasets are available from the corresponding author upon request. All data analyzed in this study are included in this published article.
